# Multi-predatory feeding aggregations in the Norwegian Sea: prevalence and ecological importance

**DOI:** 10.1098/rstb.2025.0209

**Published:** 2026-10-15

**Authors:** Leif Nøttestad, Erling Boge, Bjørn A. Krafft

**Affiliations:** Institute of Marine Research, Bergen, Vestland, Norway

**Keywords:** open ocean, predator strategies, pelagic schooling fish, prey responses, ecosystem-based fisheries management, sustainable fisheries and conservation

## Abstract

Multi-predatory feeding aggregations (MPFAs) occur when several predator species concurrently exploit shared, spatially and temporally aggregated prey. Although MPFAs are well described in some marine ecosystems, their prevalence in the Norwegian Sea has historically been underestimated. Here, we synthesize existing biological, acoustic, visual and behavioural evidence to reinterpret classical predator–prey observations through the lens of MPFAs. Our reassessment revealed that numerous events previously described as single-predator interactions actually represent multi-species feeding assemblages involving marine mammals, large predatory fishes and seabirds. These MPFAs primarily target highly abundant pelagic schooling fishes, such as Norwegian spring-spawning herring, northeast Atlantic (NEA) mackerel, blue whiting and capelin. We compiled, to our knowledge, the first region-wide overview of documented and inferred MPFAs in the Norwegian Sea, characterizing species assemblages, ecological settings and behavioural mechanisms. We further discussed how predator physiology, group foraging strategies, prey schooling dynamics and oceanographic features interact to create conditions favourable for MPFAs. Recognizing MPFAs as common and ecologically influential phenomena has important implications for understanding food webs and ecosystem-based fisheries management. Therefore, we recommend that future research in the region explicitly incorporate MPFAs through multi-sensor observational approaches, behavioural analyses and integration into ecosystem models to better represent the functional role of predators in the NEA.

This article is part of the theme issue ‘Multi-predator feeding aggregations in pelagic waters’.

## 1. Introduction

Multi-predatory feeding aggregations (MPFAs) represent temporary, dynamic assemblages in which multiple predator species simultaneously exploit shared, spatially constrained prey resources [1,2]. Although MPFAs are increasingly recognized as key drivers of trophic dynamics in many marine ecosystems [3–5], their prevalence in the Norwegian Sea has been historically underestimated. This is largely because classical ecological studies were framed around single-predator, single-prey interactions, leading to fragmented documentation of events that, when re-evaluated, clearly constitute MPFAs. By synthesizing behavioural information, acoustics, visual observations, underwater imagery and historical survey data, we reinterpreted many such ‘classical’ predator–prey events as MPFAs. This reframing provides a more realistic understanding of how multiple predators interact with abundant pelagic fish prey in highly dynamic marine ecosystems.

 Predation by multiple predators plays a central role in structuring marine food webs by affecting the abundance, habitat use and behaviour of prey species [6–9]. Understanding MPFAs is increasingly recognized as important for sustainable fisheries management and functional marine conservation, as well as for focusing on keystone or indicator species in food web modelling approaches [10,11]. Understanding the functional role of top predators is also crucial for developing ecosystem-based management strategies [9]. Over recent decades, there has been a paradigm shift from a single-species focus to a more holistic ecosystem-based fisheries management (EBFM) [12–16]. EBFM requires explicit consideration of interactions between exploited populations and their surrounding environment. However, the transition from single-species to ecosystem-level approaches involving MPFAs remains a major challenge [17]. This is partly owing to the complexity and variability of predator–prey dynamics, the spectre of attack and avoidance strategies in predators and schooling prey, especially in systems where multiple predators interact with shared aggregated prey in unpredictable ways [10,18,19]. By integrating the behavioural and spatial dynamics of MPFAs, management strategies may more effectively account for the ecological roles of key species and support more holistic, ecosystem-informed decision-making [9]. Such an approach is particularly relevant in the northeast Atlantic (NEA), including the Norwegian Sea ecosystem, which hosts some of the largest pelagic fish stocks in the world [20,21]. Although the unpredictability of MPFAs complicates management and conservation measures, adaptive management in space and time may offer improved operational and effective solutions [22,23].

## 2. Major objectives

This review synthesizes current knowledge, including revisiting and re-examining classical predator–prey interactions in the light of MPFAs, on the occurrence, prevalence and ecological significance of MPFAs in the Norwegian Sea ecosystem and adjacent waters. Our major objectives were to:

— provide a brief and overall overview of the physical, biological and trophic structure of the productive Norwegian Sea ecosystem, emphasizing its role as a key oceanic habitat for highly abundant and migratory pelagic planktivorous fish species and their multiple predators;
— describe the key predators and prey species involved in MPFAs;
— provide case studies of MPFAs in the Norwegian Sea and adjacent regions, highlighting behavioural strategies, spatiotemporal patterns and interspecific relationships; and
— discuss how improved knowledge of MPFAs may affect management and conservation within the Norwegian Sea ecosystem.

## 3. The Norwegian Sea

The Norwegian Sea is located northwest of Norway between 62° and 75° N, covering an area of approximately 1.1 million km^2^, with an average depth of 1800 m [24]. The Norwegian Sea is a large, deep, nutrient-rich, productive and open marine ecosystem [25], comprising two basins deeper than 3000 m: the Lofoten Basin in the north and the Norwegian Basin in the south, with major oceanic current systems such as the Norwegian Atlantic Current (NwAC) (figures 1 and 2). The Norwegian Sea is dominated by mesozooplankton (copepods), with an estimated zooplankton biomass in the order of 300 million tonnes, followed by macrozooplankton, such as krill (approximately 50 million tonnes) and amphipods (approximately 40 million tonnes), which are also abundant within this large marine ecosystem [24,25].

**Figure 1.**
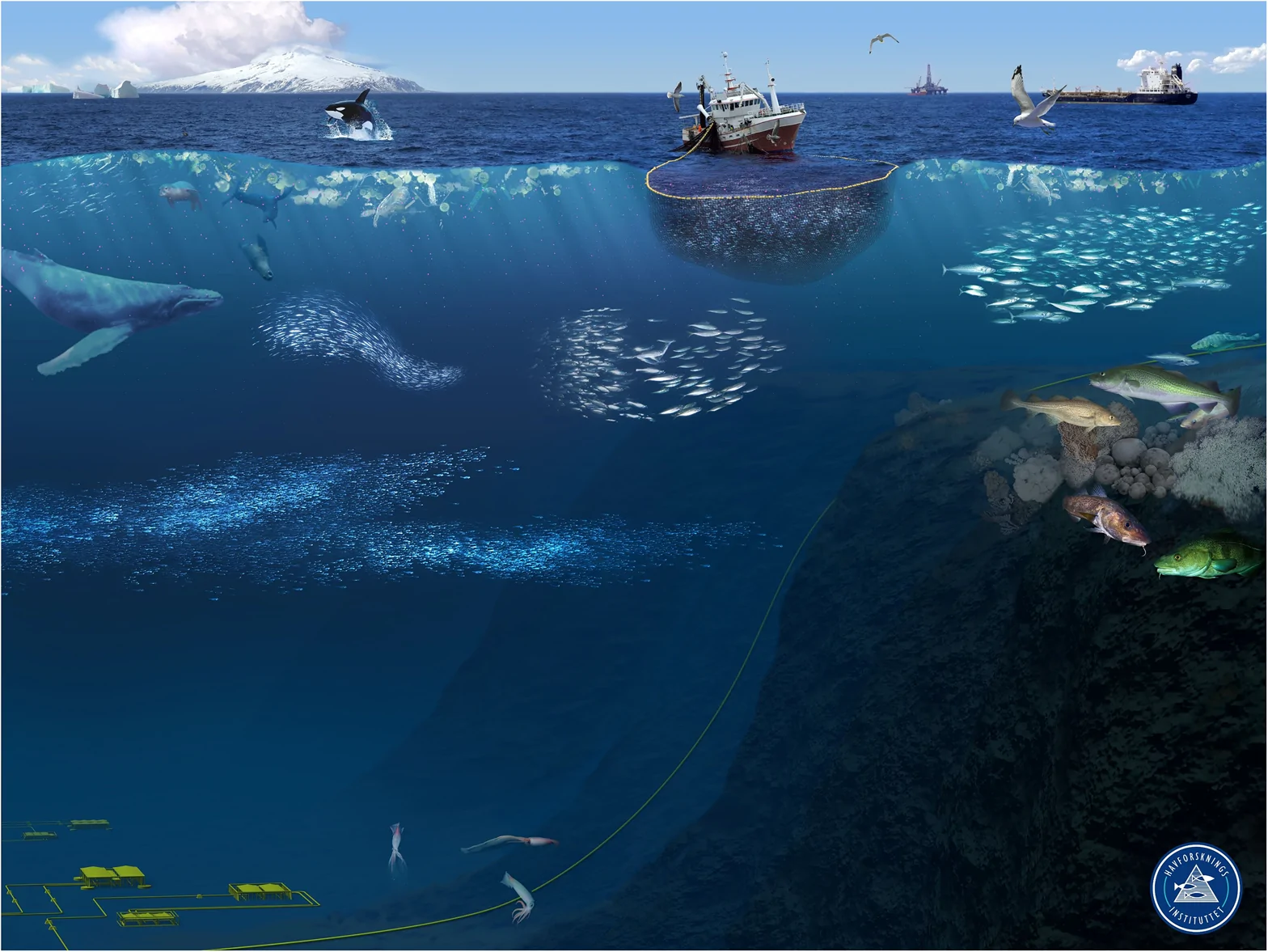
Schematic illustration of the Norwegian Sea ecosystem, including major ecologically important species at different trophic levels and human activities such as purse seine fishing, shipping and transportation, and offshore oil and gas exploration. Illustration: Institute of Marine Research, Bergen, Norway.

**Figure 2.**
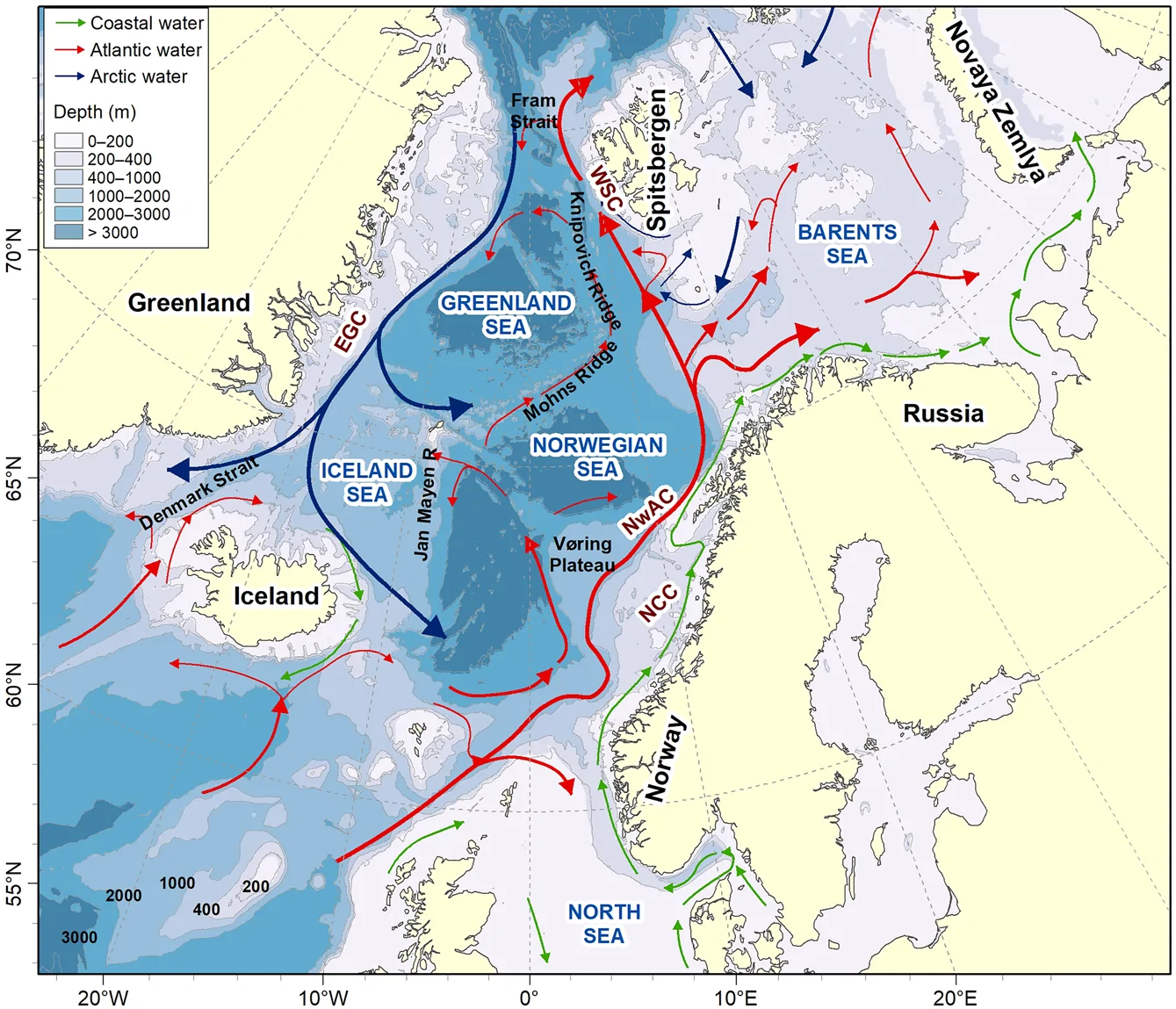
Map of the Norwegian Sea and the surrounding waters in the northeast Atlantic and in the Arctic Ocean, focusing on the major current systems, water masses (Atlantic water: blue, Arctic water: red, and coastal water: green, in the region) and topographical features. Abbreviations: NAC, North Atlantic Current; NwAC, Norwegian Atlantic Current; NCC, North Cape Current; WSC, West Spitsbergen Current; ECG, East Greenland Current. Reproduced with permission from and courtesy of Kjell Bakkeplass, Institute of Marine Research, Norway.

## 4. Key prey species in multi-predatory feeding aggregations

This review focuses primarily on the most abundant as well as ecologically and economically important small pelagic schooling fish species in the Norwegian Sea, particularly herring (*Clupea harengus)* and mackerel (*Scomber scombrus*), as key prey species involved in MPFAs. These species play a central role in energy transfer from lower to higher trophic levels, characterizing the Norwegian Sea as a ‘wasp-waist’ ecosystem dominated by mid-trophic level pelagic fish species [17,25,26]. Despite their ecological importance, model results suggest limited top-down control on planktonic prey biomass in the region [11,17]. The Norwegian Sea supports some of the world's largest managed pelagic fish stock fisheries, including Norwegian spring-spawning herring (NSSH), NEA mackerel and blue whiting (*Micromesistius poutassou*), with a combined spawning stock biomass (SSB) of between 10 and 20 million tonnes over the past decades [20,21]. These species feed primarily on mesozooplankton, krill and amphipods [25,27,28].

### (a) Norwegian spring-spawning herring

The NSSH is a cornerstone species in, and the adult part of the stock is confined within, the Norwegian Sea ecosystem ([27,29–32]). NSSH represents by far the largest herring stock in the world and historically, spawning biomass has been estimated at up to 16 million tonnes [33]. The population was estimated to be approximately 3.0 million tonnes in 2025 ([21].

NSSH has shown substantial variations over time in stock size and spatial distribution, as well as shifts during feeding, overwintering and spawning periods [29–33].

### (b) Northeast Atlantic mackerel

NEA mackerel is an abundant and highly migratory planktivorous fish in the Norwegian Sea [34]. They are both ecologically and commercially important, and are found throughout the NEA, ranging from the Iberian Peninsula to the northern Norwegian Sea, as far north as Svalbard [35,36]. Known for their schooling behaviour and rapid swimming, they primarily feed on zooplankton and small fishes in the upper 30 m of the water column above the thermocline during summer [37]. Adult mackerel undertake extensive seasonal migration driven by variations in prey availability and oceanographic conditions in the Norwegian Sea ecosystem [34,37,38]. The historical high population size of an estimated 13 million tonnes and vast geographical expansion and distribution of mackerel until 2014 influenced multiple predator–prey interactions [21,36,39,40]. This development was primarily driven by an increasing stock size but was constrained by the availability of preferred thermal habitats and prey abundance [38]. The population has been greatly and constantly reduced over the last decade and was estimated at approximately 2.7 million tonnes in 2025 [21,36].

### (c) Blue whiting

Blue whiting is a highly abundant migratory gadoid species that plays a critical role in the pelagic ecosystem of the Norwegian Sea, which serves as one of its most important feeding and nursery areas [41]. It occupies mainly mesopelagic habitats and is usually found at depths of 100–600 metres [42]. Adults migrate annually from feeding grounds in the Norwegian Sea to spawning areas along the continental shelf edge west of the British Isles (mainly from February to April) [43]. They feed on zooplankton, particularly krill and amphipods, and smaller fishes, as well as occasionally competing for food with herring and mackerel [27,28], and are targeted by a range of predators [43]. The population was estimated to be approximately 4.5 million tonnes in 2025 [41].

### (d) Capelin

Capelin (*Mallotus villosus*) in the eastern part of the Norwegian Sea, primarily representing the Barents Sea stock, is a small, short-lived pelagic fish that serves as a cornerstone species of the Arctic marine ecosystem [44]. It acts as a key forage fish, converting plankton into energy for predators such as northeast Arctic cod, marine mammals and seabirds [45]. The mature part of the capelin population migrates towards the coast in January and February and typically reaches land at the beginning of March. They then spawn along the coasts of northern Norway in the Troms and Finnmark counties, and the Kola Peninsula in Russia. Most individuals die after spawning, usually when they are three or four years old. The population size has varied enormously over time, from a historically high biomass of approximately 5–6 million tonnes in the mid-1970s and 1991 to a present low biomass of approximately 340 000 tonnes capelin in 2025 [46].

## 5. Key predator species in multi-predator feeding aggregations

The Norwegian Sea ecosystem supports a diverse assemblage of marine predators that may participate in MPFAs [31,47]. These aggregations are shaped by the morphological traits and energetic demands of the species involved, which influence their foraging strategies, spatial overlap and temporal synchrony with prey availability. Beyond morphological traits and energy demands, predator and prey behaviour and physiology strongly shape the likelihood and form of MPFAs. Factors such as sociality, group coordination, swimming performance, diving capacity, air‑breathing constraints and sensory modalities influence how predators can synchronize attacks and exploit ephemeral prey patches. Similarly, prey schooling behaviour, escape responses and vertical migration patterns determine how vulnerable they are to simultaneous exploitation [48]. Incorporating these traits provides a mechanistic understanding of why certain predator assemblages recur across MPFAs in the Norwegian Sea.

### (a) Marine mammals

The Norwegian Sea is an important migration corridor and active feeding area for several marine mammals [11,47,49], including fin (*Balaenoptera physalus*)-, minke (*Balaenoptera acutorostrata*)-, humpback * (Megaptera novaeangliae*)-, pilot (*Globicephala melas*)- and killer whales (*Orcinus orca*) [39,40,47,50,51]. Summer population sizes in these waters are estimated to include approximately 10 000 fin whales, approximately 10 000 humpback whales, approximately 87 000 long-finned pilot whales and approximately 15 000 killer whales [50]. Marine mammals in the Norwegian Sea are estimated to consume an average of 3.1 (CI: 1.3-5.9) million tonnes of fish annually [49], which is significantly more than the annual average fisheries catch, which is in the order of 1-1.5 million tonnes. Marine mammals, such as group-foraging humpback whales and fin whales, pods of killer whales, pilot whales and various dolphin species, are occasionally involved in MPFAs, often engaging in coordinated and/or opportunistic feeding behaviour [31,39,40,47,52–54].

### (b) Predatory fishes

Major predatory fish species feeding on planktivorous fishes in the Norwegian Sea and surrounding waters are relatively few and probably constitutes around 1 million tonnes, dominated by NEA saithe (*Pollachius virens*) and northeast Arctic cod (*Godus morhua*) [31,55,56]. Atlantic bluefin tuna (BFT) (*Thunnus thynnus*) has become, after its comeback to Norwegian waters in 2012, an increasingly important ecological player and top predator on pelagic planktivorous fish species along the coast of Norway and in the Norwegian Sea during the last decade [57,58]. The total SSB, although unknown, may be approximately 500 000 tonnes [59].

### (c) Seabirds

The Norwegian Sea is also an important area for some of the largest seabird populations in the NEA [60]. The Atlantic puffin (*Fractercula arcica*) used to have, in the early 1980s, a large population size (1.8 million pairs) in the Norwegian Sea [60] and actively feeds on fish larvae and juvenile fishes [61]. A substantial decline in breeding puffins has been documented over several decades to around 225 000 pairs [62]. Similarly, black-legged kittiwakes (*Rissa tridactyla)* have also experienced a substantial decline from around 250 000 pairs to less than 50 000 pairs over several decades since the early 1980s [62]. The black-legged kittiwake is the most numerous species of gull in the world and is the most oceanic in its habits. The northern fulmar (*Fulmarus glacialis*), with some 500 000 pairs in the Norwegian Sea, is an interesting and adaptive seabird species opportunistically involved in MPFAs within the Norwegian Sea [62]. There are approximately 3300 pairs of northern gannets (*Morus bassanus*). The seabirds in the Norwegian Sea may consume approximately 1.24 million tonnes of marine food items annually [60], of which a considerable amount consist of early life stages of pelagic planktivorous fish species and particularly NSSH [62,63]. Norway also holds the largest breeding population of white-tailed eagles (*Haliaeetus albicilla*) in Europe [64], which has increased significantly over the last decade. They are occasionally involved in MPFA, preying on a variety of pelagic fish and seabird species [64,65].

## 6. Overview of multi-predator feeding aggregation in the Norwegian Sea

An overview of the observed and documented MPFAs in the Norwegian Sea and surrounding waters is presented in table 1. The northerly shift in the distribution of marine mammals, such as fin whales, humpback whales and killer whales, is strongly associated with the northerly shift in the geographical distribution of major prey species, such as NSSH and NEA mackerel, in the Norwegian Sea and surrounding waters. These large-scale changes documented over the last few decades have created, for example, feeding hotspots for different top predators in MPFAs, which may involve marine mammals, large predatory fishes and seabirds, caused by different driving forces and mechanisms ( table 1; [32,39,50,62,69–71]). The MPFAs listed in table 1 involve spectacular multiple species interactions, where different predator strategies and anti-predator schooling behaviour may be studied and revealed simultaneously. Pronounced annual long distance migration patterns and schooling dynamics over time for the most abundant planktivorous fish species appearing in the Norwegian Sea seem to reflect the observed adaptive predator strategies of the different predators involved in our MPFAs ([31,40,52,55]). Coordinated group hunting and foraging dominated our MPFAs case studies involving marine mammals, such as killer whales, fin whales, humpback whales, white-sided dolphins and long-finned pilot whales ( table 1 and figure 3a). The only exception was the large blue whale feeding on NSSH together with a pod of killer whales off the western part of Vesterålen, northern Norway during winter ( figure 3b).

**Table 1.** Selected case studies of MPFAs in the Norwegian Sea and surrounding oceanic and coastal waters.

predator species	prey species	situation	references
humpback whales, killer whales, NEA cod, NEA saithe, white-tailed eagle, seagulls, kittiwakes, northern fulmar	NSSH	overwintering areas in coastal waters and within narrow fjords in northern Norway	[31,47,52,53,55]
humpback whales, northern fulmar, kittiwakes, seagulls	Capelin	summer feeding period off islands (Jan Mayen, Bear Island, Svalbard)	[66,67]
fin whales, white-sided dolphins, seagulls	NSSH	spring feeding period in offshore waters	[39,55]
fin whales, northern fulmar	NSSH	summer feeding in shelf area between Svalbard and Norway	[66,67]
killer whales, northern gannets, northern fulmar, kittiwakes, seagulls	NEA mackerel	summer feeding in offshore waters	[40,57]
long-finned pilot whales, northern fulmar, seagulls	blue whiting	summer feeding in southern Norwegian Sea	[39,66]
NEA cod, NEA saithe	NSSH	spring feeding along continental shelf and in coastal waters	[56,67]
Atlantic bluefin tuna, northern gannets, seagulls	NEA mackerel	summer feeding in coastal waters	[57,58,68]

**Figure 3.**
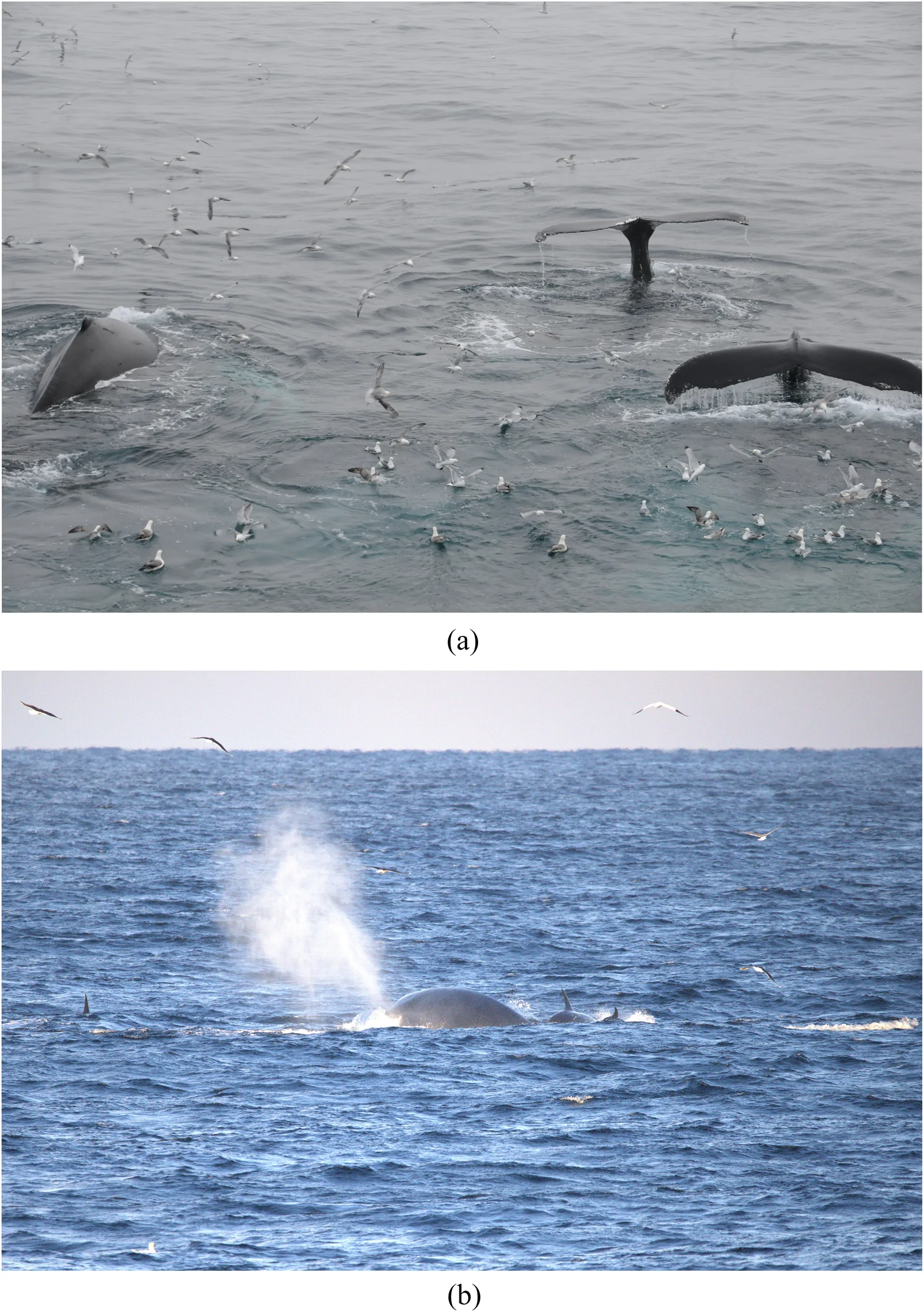
(a) MPFAs with groups of humpback whales and numerous seabirds, such as northern fulmars and kittiwakes, hunting for and feeding on capelin during night-time in mid-summer, east of the island Jan Mayen, in the northwestern part of the Norwegian Sea [39]. Photo: Leif Nøttestad, Institute of Marine Research, Norway. (b) Blue whale, pod of killer whales, northern gannets and seagulls hunting for and foraging on schools of NSSH recorded acoustically [63] in the western part of Vesterålen, northern Norway in wintertime. Photo: Erling Boge, Institute of Marine Research, Norway.

Large predatory fishes involved in our case studies of MPFAs either aggregated and forced their schooling prey into denser schools and closer to the surface or took advantage of larger BFT that have been involved in MPFAs after their recent comeback to Norwegian waters, particularly along the west coast of Norway when targeting and preying on schooling mackerel of various age groups, accompanied by opportunistic seabirds such as northern gannets (*Morus bassanus*) and seagulls. Seabirds take advantage of BFT aggregating the mackerel schools of different size groups into dense concentrations close to the surface (see table 1) [68].

## 7. Discussion

The ecological role of MPFAs in marine pelagic ecosystems varies considerably, reflecting differences in trophic structure, from bottom-up and wasp-waist systems to top-down controlled ecosystems [11,17,25,26]). In the Norwegian Sea, MPFAs are spatially and temporally dynamic [24,39]. Surprisingly, few direct studies have been documented in the existing literature. Nevertheless, after re-examining the available literature in more detail, MPFAs could be found and revealed. However, this available information was not previously presented in the context of MPFAs. By revisiting additional available information, such as aerial and underwater photos and acoustic recordings (sonar and echosounder) from surveys, we have realized that the actual prevalence of MPFAs in the Norwegian Sea must be substantially higher than that suggested in the existing scientific literature. A major reason is that ecological studies have previously been analysed within the framework of classical single species predator–prey interactions, involving one dominant predator foraging on specific prey species, rather than focusing on MPFAs *per se.* MPFAs in the Norwegian Sea appear to emerge from the interaction of several ecological mechanisms: (i) environmental drivers, such as oceanic fronts, eddies and topographic features that create transient prey hotspots [72,73]; (ii) spatial and temporal overlap between migratory predators and seasonally aggregated pelagic prey [69,74]; (iii) facilitative interactions among predators that increase foraging efficiency [75]; and (iv) behaviourally mediated responses by prey that concentrate biomass near the surface [76]. These mechanisms differ in predictability between the Norwegian Sea and, for example, the Southern Ocean, where krill swarms form consistent, high‑density prey patches [77,]. By contrast, the Norwegian Sea is characterized by more mobile, migratory and dynamic landscape‑scale prey fields, leading to more ephemeral and context‑dependent MPFAs [24,74]. Recognizing these coupled dynamics is essential for incorporating MPFAs into ecosystem-based management frameworks.

The contrast between the Norwegian Sea in the far north, close to the Arctic, and the Southern Ocean in the far south, close to Antarctica, provides an important ecological context for understanding MPFAs. The Southern Ocean represents a more geographically isolated marine system, shaped by the Antarctic Circumpolar Current and a relatively closed subpolar gyre structure [78,79]. This physical isolation has produced a food web characterized by lower species diversity but dominated by a single, phylogenetically distinct and super‑abundant mid-trophic species, Antarctic krill, which functionally replaces the roles of both small pelagic fish and mesozooplankton typical of northern ecosystems [80,81].

By contrast, the Norwegian Sea is a more open and advective ecosystem with high biodiversity, a strong inflow of Atlantic water and multiple coexisting pelagic fish species that together occupy the mid-trophic niche [24,25,28]. These differences shape the expression of MPFAs: in the Southern Ocean, predictable krill hotspots support recurrent, multi‑predator feeding aggregations [82], whereas in the Norwegian Sea, MPFAs emerge from more dynamic, mobile and migratory landscape‑scale prey fields [69,74], including unpredictable foraging situations [83]. However, until now, there has been no case of MPFA in the Norwegian Sea listed. Management paradigms also diverge markedly. Southern Ocean fisheries operate under a predator‑first precautionary framework, ensuring that krill harvests do not compromise the needs of dependent predators [84,85]. In the Norwegian Sea, management has traditionally been more harvest centred, with less explicit integration of predator–prey constraints [70,86]. Recognizing these structural differences is essential for understanding why MPFAs manifest differently across ocean basins and how ecosystem‑based management must be tailored accordingly.

MPFAs in the Southern Ocean are better described, largely owing to the presence of super-abundant Antarctic krill swarms, which serve as a keystone prey species for a wide range of predators [4,87]. These krill aggregations often occur in predictable hotspots, both spatially and seasonally, facilitating repeated observation and targeted research. The consistent availability of a single, high-density prey resource supports simultaneous foraging by multiple predator species, making the Southern Ocean an ideal natural laboratory for studying MPFAs and their ecological dynamics.

The limited number of studies so far emphasized on MPFAs in the Norwegian Sea may partly be owing to the dispersed and highly dynamic schooling behaviour over extensive geographical areas during the active feeding period from spring to autumn for the behaviour of key planktivorous fish species, such as mackerel, herring, blue whiting and capelin [27,37,39,88,89]. Nevertheless, the major reason is probably linked to the fact that previous studies in the Norwegian Sea did not focus on MPFAs, rather focusing solely on selected major single species and more traditional predator–prey interactions [31].

Pelagic schooling species typically form smaller, less dense schools during the active feeding period to optimize prey intake and reduce intraspecific competition [37,90,91]. The vast Norwegian Sea ecosystem lacks, to some extent, consistent and predictable spatial and temporal prey hotspots, making MPFAs difficult to detect and quantify with advanced underwater acoustics, high-resolution camera technology and visual observations in the open ocean [47]. The offshore regions of the Norwegian Sea serve as a vital migratory corridor for many large marine mammals, including large baleen whales that travel seasonally between tropical breeding grounds and Arctic feeding areas [11,69]. These species typically pass through the region during their northward migration in spring and southward return in autumn. As a result, their presence in the Norwegian Sea is often brief and transitional, with limited time spent foraging in the area compared with more productive and predictable Arctic zones further north. Thus, simultaneous observations of multiple predators and prey can be very challenging in the open ocean owing to the more unpredictable nature of such interactions, which poses substantial challenges for integrating results from quantitative MPFAs into operational fisheries management and conservation frameworks and application.

The most predictable MPFAs in the Norwegian Sea are assumed to occur north of the Arctic Circle, along the ice edge, especially during spring and summer, around Jan Mayen, Bear Island and along the ridge from North Cape to Svalbard [39,40,50,54]. These areas feature steep topography and complex ocean circulation and mixing patterns of Atlantic and Arctic water masses, which promote regional upwelling and increased productivity [92]. Such conditions attract dense schools of herring and capelin feeding on zooplankton and krill, in turn drawing top predators like humpback and fin whales [31,39,50], often accompanied by coastal and oceanic seabirds [60].

### (a) Predator interactions in multi-predatory feeding aggregations

Most MPFAs in the Norwegian Sea and adjacent oceanic and coastal areas seem to be a combination of group-foraging marine mammals, larger fish species and/or seabirds (figures 1 and 3a–b; table 1). These species act as multiple predators feeding on highly abundant pelagic planktivorous and schooling/shoaling fish species, such as herring, mackerel, blue whiting and capelin in different life stages and size groups throughout the Norwegian Sea and surrounding oceanic and coastal waters [11,17,24,31,36,39–41,49,53–55,57,60,64,69,93,94]. Furthermore, from a more behavioural and ecological perspective, it is possible to reveal and quantify sophisticated predator hunting strategies and corresponding antipredator behaviour simultaneously using multi-beam sonars, echo-sounders and underwater cameras in combination with visual observations [47,55,61]. In addition to ship‑based hydroacoustics, multi-beam sonars, underwater cameras and visual observations, several emerging technologies offer new possibilities for quantifying MPFAs. Biologging tags provide high‑resolution information on predator movement and foraging behaviour at fine temporal scales [95,96], whereas drones and camera systems enable non‑invasive, near‑surface behavioural observations of coordinated predator–prey interactions [97–99]. These tools, used increasingly in the Norwegian Sea and comparable ecosystems, have already revealed predator synchrony, prey herding and multi‑species facilitation dynamics that would otherwise remain undetected [52].

The present overwintering areas with dense aggregations of schooling herring (NSSH) close to the coast and even inside spatially constrained and narrow fjord systems in northern Norway [94] have also created a haven for multi-predator feeding aggregations (e.g. [31,47,53]). This highly rich and geographically restricted marine ecosystem, concentrated around super-aggregated herring schools easily accessible at short distances from land, may be sustained for several months during autumn and winter. This spatial redistribution of both overwintering and spawning aggregations of herring [32] has altered and shifted multiple predator–prey dynamics and interspecific interactions across regions and seasons.

Schooling pelagic fish species that feed on zooplankton prey may also create MPFAs with seabirds. When multi-predatory feeding events occur, they are often initiated by the largest predators, such as humpback whales [39,47,54], fin whales [39] and killer whales [31,40,53,55,100,101], followed and accompanied by larger predatory fish species, such as cod and saithe [31,47,55]. When schooling prey species are forced and trapped towards the surface or into shallower waters, various seabird species may be attracted to the scene and take advantage of the occurred prey availability [102], owing to other group-hunting predators deliberately forcing and trapping the prey into shallow water and/or close to the surface [55]. This is prevalent for shallow-diving seabirds, such as seagulls (*Larus* sp.) and white-tailed sea eagles [31,64,65].

A major ecological change during the last decade is the comeback, significantly increased abundance, northern distribution and establishment of Atlantic BFT within the Norwegian Sea and adjacent waters [57,58]. This has led to an increased ecological impact and predation from BFT, as a top predator, particularly on abundant and commercially important fish species, such as mackerel and herring [68]. This has implications for the future sustainable management and conservation of both small-pelagic fish species and BFT in their respective marine ecosystems [103]. During the last decade, BFT have been increasingly observed to be involved in MPFAs by fishermen and during citizen science. BFT feed on various schooling fish and other prey in Norwegian waters, primarily during their annual feeding migrations. Mackerel and herring are key components of their diet [57,104]. Interestingly, BFT targets fish of different sizes and life stages, such as mackerel, ranging from small juveniles to large adults [68].

The most spectacular and striking MPFAs found in Norwegian waters take place annually during the massive overwintering period, counting billions of herring heavily concentrated in large and dense schools and aggregations ([31,32,47,105]. MPFAs have been observed and documented in late autumn and winter along the northern coasts and inside the narrow fjords of Norway. These intense feeding events involve multiple predator species, including marine mammals, predatory fishes and numerous seabird species. Addressing this complexity is important not only for the long-term sustainability and conservation of marine species but also for enhancing ecological understanding for improved EBFM management.

## 8. Conclusion

The Norwegian Sea is a highly productive and dynamic marine ecosystem, supporting an estimated 300 million tonnes (approx.) of zooplankton, 10–20 million tonnes (approx.) of key pelagic fish species, 1 million tonnes (approx.) of pelagic predatory fishes, 200 000 (approx.) individual marine mammals and 4.5 million (approx.) seabirds. Our review strongly suggests that the prevalence and ecological significance of MPFAs are much higher than previously assumed and documented, where highly abundant pelagic planktivorous fish schooling species, serve as dominant prey for multiple predators of marine mammals, large predatory fish species and numerous seabird species. MPFAs must be considerably more prevalent than suggested in available scientific literature on the Norwegian Sea ecosystem and surrounding waters. Therefore, it is recommended that future ecological studies focus more systematically on all involved players during MPFAs and not only on selected and classical single predator–prey interactions, using state-of-the-art and complementary underwater and aerial technologies at different scales and spatial resolutions. This may indeed increase our overall understanding and knowledge of the prevalence and importance of MPFAs within the Norwegian Sea ecosystem to be sufficiently included and implemented in future fish stock assessments, food web and ecological models to improve our long-term management and conservation of valuable marine species. Further improving the delicate balance between operational fisheries management and effective marine conservation in a changing environment remains essential.

## Supplementary Material



## Data Availability

Supplementary material is available online [106].

## Theme

One contribution of 14 to a theme issue ‘Multi predator feeding aggregations in pelagic waters’.
