# Supplementary material for: Multi-predatory feeding aggregations in the Norwegian Sea: prevalence and ecological importance
Source: Philos Trans R Soc Lond B Biol Sci. 2026 Oct 15;381(1961):20250209. doi: 10.1098/rstb.2025.0209 (PMC13670076; doi:10.1098/rstb.2025.0209)
Supplement: Supplementary file 1 [file rstb.2025.0209.zip › rstb.2025.0209/rstb20250209_si_001.docx]

**Supplementary**

*Supplementary Table 1****.*** Overview of major top-predator species of marine mammals, fish and seabirds versus dominant pelagic planktivorous prey species in the Norwegian Sea ecosystem. Population sizes are provided as individuals for marine mammals and seabirds, tonnes for Atlantic bluefin tuna as top predator, and tonnes for the most abundant pelagic prey species in the Northeast Atlantic and Norwegian Sea (Huse et al., 2014; Shimmings and Øien, 2015; Leonard and Øien, 2020; ICCAT, 2022; ICES, 2025a, b, c; Bogstad, 2025; NAMMCO, 2025; SEAPOP 2025; Fauchald and Vang, 2025).

| **Species** | **Latin name** | **Population size** | **Units** |
| --- | --- | --- | --- |
| **Marine mammals** |  |  |  |
| Humpback whales | *Megaptera novaeangliae* | ~10 000 | individuals |
| Fin whales | *Balaenoptera physalus* | ~10 000 | individuals |
| Long-finned pilot whales | Globicephala melas | ~87 000 | individuals |
| Killer whales | *Orcinus orca* | ~15 000 | individuals |
| **Fish species** |  |  |  |
| Atlantic bluefin tuna | *Thunnus thynnus* | ~500 000 | tonnes |
| NEA mackerel | *Scomber scombrus* | ~2 740 000 | tonnes |
| NSS herring | *Clupea harengus* | ~3 000 000 | tonnes |
| Northeast Arctic cod | *Gadus morhua* | ~550 000 | tonnes |
| Northeast Arctic saithe | *Pollachius virens* | ~400 000 | tonnes |
| Atlantic blue whiting | *Micromesistius poutassou* | ~4 500 000 | tonnes |
| **Seabirds** |  |  |  |
| Northern gannet | *Morus bassanus* | ~3 600 | individuals |
| Northern fulmar | *Fulmarus glacialis* | ~1000000 | individuals |
| Black-legged kittiwake | *Rissa tridactyla* | ~150 000* | individuals |
| Sea Gulls | *Larus spp.* | ~200 000 | individuals |
| Atlantic puffin | *Fratercula arctica* | ~553 000 | individuals |
| Common guillemot | *Uria aalgia* | ~30 000 | individuals |
| White-tailed sea eagle | *Haliaeetus albicilla* | >5 600 | individuals |
